# Efficient molasses utilization for low-molecular-weight poly-γ-glutamic acid production using a novel *Bacillus subtilis* stain

**DOI:** 10.1186/s12934-022-01867-5

**Published:** 2022-07-16

**Authors:** Jing Li, Shengbao Chen, Jiaming Fu, Jianchun Xie, Jiansong Ju, Bo Yu, Limin Wang

**Affiliations:** 1 Beijing Advanced Innovation Center for Food Nutrition and Human Health, Beijing Technology & Business University (BTBU), Beijing, 100048 People’s Republic of China; 2 College of Life Science, Hebei Normal University, Shijiazhuang, 050024 People’s Republic of China; 3 CAS Key Laboratory of Microbial Physiological & Metabolic Engineering, State Key Laboratory of Mycology, Institute of Microbiology, Chinese Academy of Sciences, Beijing, 100101 China; 4 College of Chemical Engineering, Nanjing Forestry University, Nanjing, 210037 People’s Republic of China

**Keywords:** Poly-γ-glutamic acid, *Bacillus subtilis*, Cane molasses, Low molecular weight

## Abstract

**Background:**

Poly-γ-glutamic acid (γ-PGA) is a biopolymer and has various applications based on its biocompatibility, non-toxicity, and edibility. Low-molecular-weight (Mw)-γ-PGA has promising applications in agriculture and pharmaceuticals. It is traditionally produced by enzymatic hydrolysis. Cost-effective bioproduction of low-Mw-γ-PGA is essential for commercial application of γ-PGA.

**Results:**

*Bacillus subtilis* 242 is a newly isolated low-Mw-γ-PGA-producing strain. To develop cost-effective production of γ-PGA using this newly isolated strain, cane molasses and corn steep liquor were used to produce γ-PGA. The concentration of cane molasses was optimized and 100 g/L cane molasses resulted in high γ-PGA production. The effects of yeast extract and corn steep liquor on γ-PGA yield were investigated. High concentration of γ-PGA was obtained in the medium with corn steep liquor. A concentration of 32.14 g/L γ-PGA was achieved in fed-batch fermentation, with a productivity of 0.67 g/L/h and a percentage yield (*g*_γ-PGA_/*g*_glutamate_) of 106.39%. The Mw of γ-PGA was 27.99 kDa.

**Conclusion:**

This study demonstrated the potential application of *B. subtilis* 242 for cost-effective production of low-Mw-γ-PGA from cane molasses.

## Background

Natural biopolymers have attracted extensive interest owing to their environmental friendship. As a result of increasing environmental concerns, bioproduction of polymers using microorganisms has been especially developed [1, 2]. Poly-γ-glutamic acid (γ-PGA) is a natural biopolymer composed of glutamic acid monomers with **γ**-amide linkage [3]. γ-PGA and poly-ε-lysine (ε-PL) are two biopolymers that could be synthesized by microorganisms [4]. γ-PGA is water soluble, biodegradable, edible, and environmentally friendly, and it has various applications in medicine, foods, plastics, water treatment, and agriculture [2, 5].

γ-PGA is the component of the traditional Japanese food *natto*, made from soy beans fermented by *Bacillus* strains [6]. Several *Bacillus* strains, including *B. subtilis* natto, *B. subtilis* chungkookjang, *B. licheniformis*, and *B. amyloliquefaciens*, have been reported to produce γ-PGA outside cells, and *Bacillus* species are producers for commercial production of γ-PGA [3, 7–9]. Up to now, intensive studies regarding γ-PGA fermentation, synthetic mechanism and metabolic engineering have been carried out to improve γ-PGA yield [10–12]. Low-cost substrates and efficient strains are essential for the commercial uses for γ-PGA. Researchers have been focused on improving the economic feasibility of γ-PGA fermentation by using cost-effective materials. Jerusalem artichoke, rice straw, glycerol, and sucrose have been used to replace glucose for γ-PGA production [13–15]. Cane molasses is a by-product of sugar refinery [16]. The annual production of cane molasses is approximately 3 million tons per year in China, which is mostly available in Guangxi province in Southern China. Cane molasses is traditionally used as feed materials or simply discharged [17]. It contains approximately 50% (w/w) sugars and a small amount of nitrogenous compounds, which could be used as inexpensive substrate for bio-based chemicals production, such as butyric acid and polyhydroxyalkanoate [17, 18]. Efficient utilization of cane molasses to produce γ-PGA would be of great significance. The second challenge of the commercial applications of γ-PGA is the production of tailor-made γ-PGA [19]. The molecular weight (Mw) differs substantially (from 10 to 1 × 10^4^ kDa) depending on the species used. Different Mws may influence the physical properties of γ-PGA, which can be exploited in different applications. High-Mw-γ-PGAs (Mw > 1 × 10^3^ kDa) have strong viscosity and could be used as thickeners or flocculant [20]. Low-Mw-γ-PGAs (Mw < 500 kDa) could be used as drug delivery (45–60 kDa), tissue engineering nanocomposites (20–275 kDa), water-retaining agents (< 20 kDa), and so on [21, 22]. At present, the Mws of commercial γ-PGA produced *Bacillus* species are approximately 1 × 10^3^ kDa, which limits the wide uses of γ-PGA [21]. Enzymatic hydrolysis, physical and chemical methods have been made to depolymerize γ-PGA [3]. However, the low yield and high cost inhibited the applications of γ-PGA.

In this study, a novel low-Mw-γ-PGA producer, *B. subtilis* 242, was used to produce γ-PGA from cane molasses. The effects of carbon and nitrogen sources on γ-PGA production were investigated. Fed-batch fermentation was performed in a 5-L fermenter to produce γ-PGA from cane molasses. The results demonstrated the potential application of *B. subtilis* 242 for economic low-Mw-γ-PGA production from a by-product of sugar refinery.

## Results and discussion

### Isolation and identification of the γ-PGA-producing strain

**Fig. 1:**
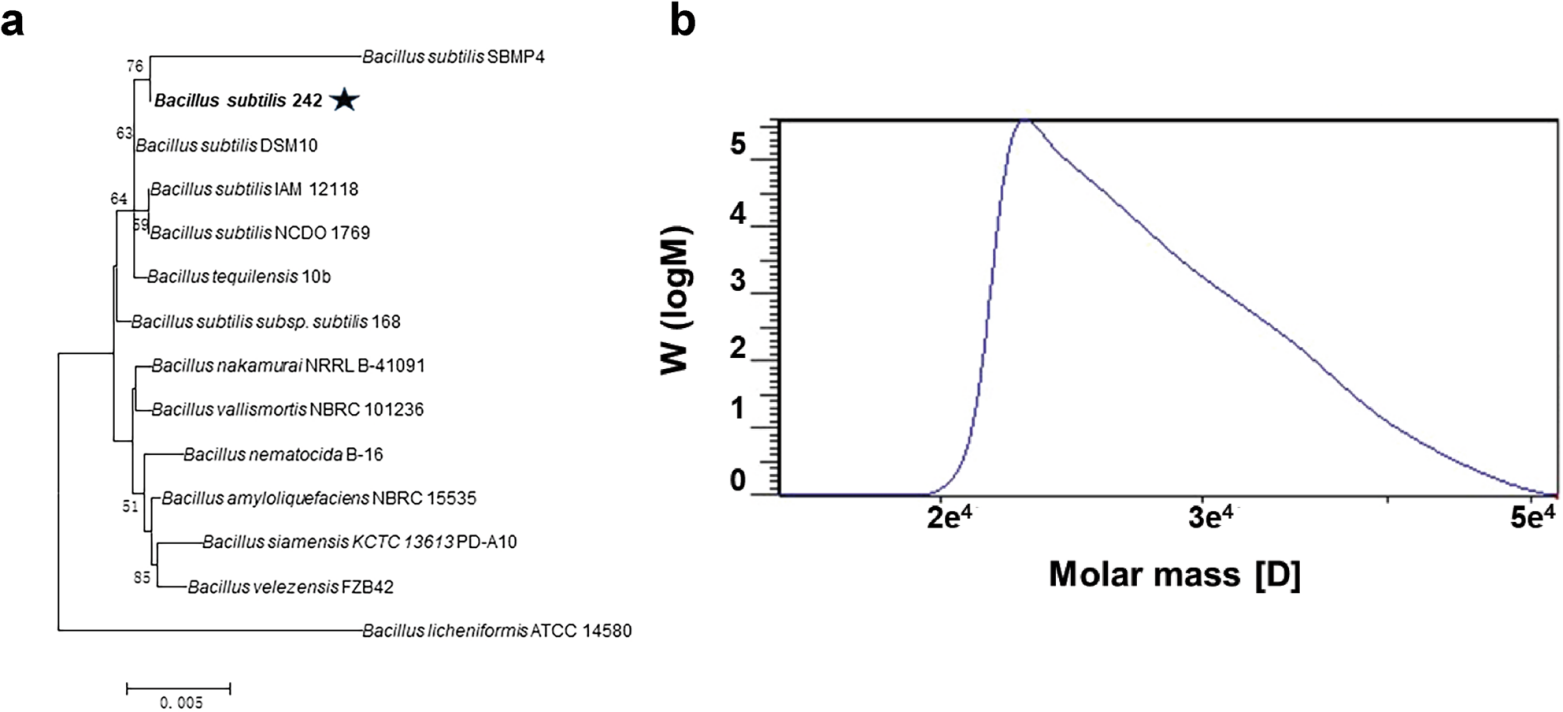
Identification and characterization of the newly isolated strain. Phylogenetic relationship of *B. subtilis* 242 and other *Bacillus* strains based on neighbor-joining tree analysis of the 16s rDNA sequence (**a**). The bar (0.005) at the bottom of the tree indicates the substitutions per nucleotide position. Molecular weight of γ-PGA produced by *B. subtilis* 242 (**b**)

To screen γ-PGA producers, 260 isolates with mucoid colonies were picked up from the agar plates with glutamate. These strains were transferred into the fermentation medium and the strain with the highest γ-PGA production was designated as strain 242. The 16s rDNA gene sequence of strain 242 showed the similarity to *B. subtilis* DSM10 (99.93%). A phylogenetic tree was constructed based on the 16s rDNA sequence. As shown in Fig. 1a, strain 242 formed a cluster with *B. subtilis* SBMP4, and was classified as the species *B. subtilis*. *B. subtilis* 242 was deposited at the China General Microbiological Culture Collection Center (CGMCC) with the Accession number CGMCC NO. 23791.

**Fig. 2:**
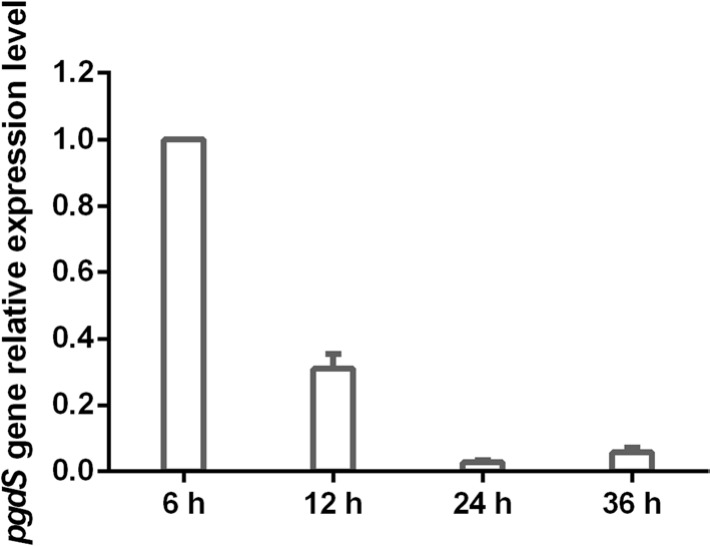
Determination of the relative transcriptional level of *pgdS* gene at different fermentation time. The threshold cycles (*C*_T_) for each PCR with different concentrations of cDNA were determined and were compared with that for a standard DNA (the 16S rRNA gene) that was also analyzed at the same time. The y-axis indicates the transcription ratio of *pgdS* at 12 h, 24 h, 36 h to 6 h. Error bars represent SD calculated from four independent determinations

Glucose and yeast extract were used as the carbon source and nitrogen source, respectively, to produce γ-PGA by the newly isolated *B. subtilis* 242. The Mw of γ-PGA was detected by gel permeation chromatography (GPC). The Mw of γ-PGA produced by *B. subtilis* 242 was 28.49 kDa (Fig. 1b), which indicated that *B. subtilis* 242 is a low-Mw-γ-PGA producer. *Bacillus* species are the main producers of γ-PGA. The Mws of γ-PGA produced by most *Bacillus* species were higher than 100 kDa [13]. PgdS (the γ-DL-glutamyl hydrolase) is an endo-type hydrolase that cleaves the γ-glutamyl bond and depolymerizes high-Mw-γ-PGA to low-Mw-γ-PGA [3]. It is the key enzyme responsible for the Mw control in γ-PGA producers [21, 22]. To investigate the reason for low-Mw-γ-PGA production by *B. subtilis* 242, the transcripts of *pgdS* at different fermentation periods (6, 12, 24, 24 and 36 h) were detected using quantitative real-time (RT)-PCR. As shown in Fig. 2, *pgdS* transcription ratios of 12 h to 6 h was 0.31 ± 0.04. Notably, the transcription ratio decreased to 0.06 ± 0.01 when cells were cultured for 36 h. High *pgdS* expression at the early period of γ-PGA fermentation play an important role in regulating Mw of γ-PGA produced by *B. subtilis* 242. PgdS may cleave γ-glutamyl bonds between glutamate residues in γ-PGA, thus producing γ-PGA with low Mw. In this study, a low-Mw-γ-PGA producer, *B. subtilis* 242, was isolated and it was used for the cost-effective production of γ-PGA.

### Characterization of cane molasses utilization by *B. subtilis* 242

**Fig. 3:**
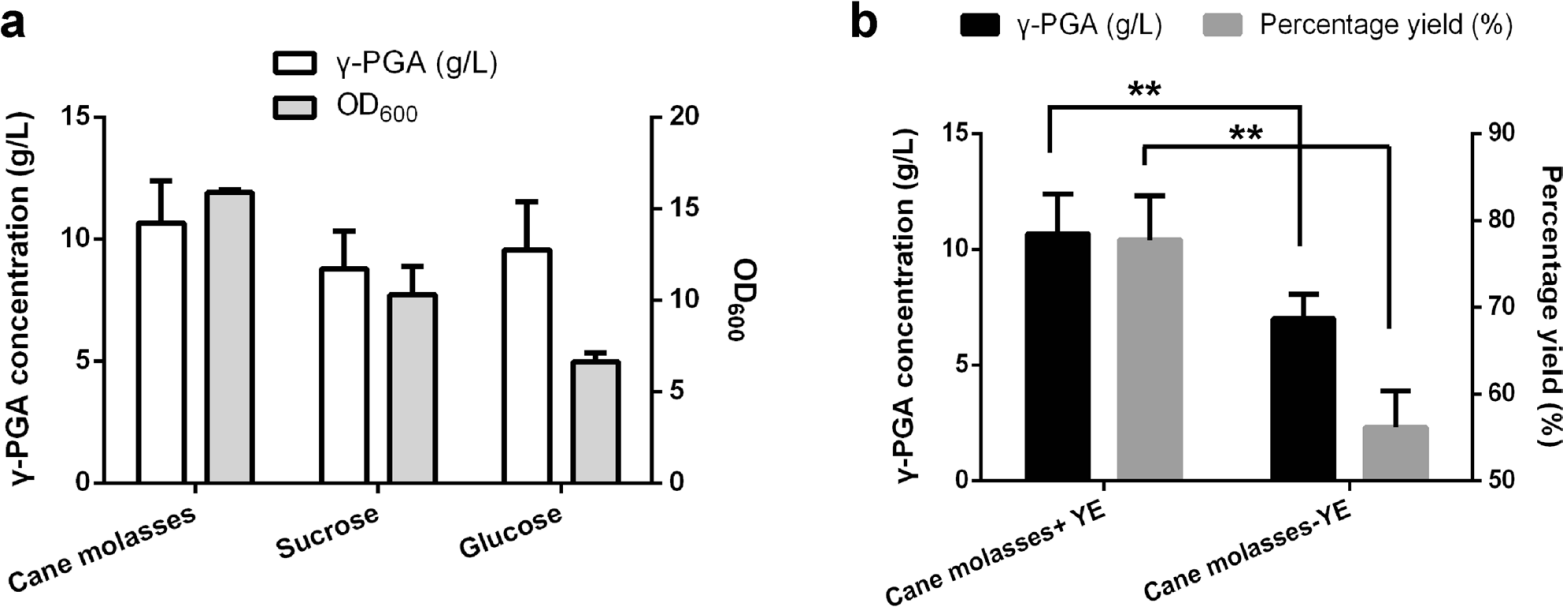
Comparison of different carbon sources (**a**) and yeast extract (**b**) for the production of γ-PGA using *B. subtilis* 242. Error bars represent SD calculated from three independent determinations. **Indicate significant differences at p < 0.05

Cane molasse is a major by-product of sugar industry. It contains approximately 50% (w/w) sugars (mainly sucrose, fructose and glucose) and a small quantity of crude protein (3%), ash (8%), metal ions (8%) and water (25%) [23]. To investigate the feasibility of cane molasses as a substrate for γ-PGA production by *B. subtilis* 242, total sugars in cane molasses were detected using high performance liquid chromatography (HPLC). Cane molasses contains 395 g/L sucrose, 3 g/L glucose and 13 g/L fructose. Same concentrations of initial sugars (~ 20 g/L) were used in shake-flask fermentations to study the effects of cane molasses, sucrose and glucose, individually, on γ-PGA production. As shown in Fig. 3a, 10.67 ± 1.73 g/L γ-PGA was obtained in the medium with cane molasses, which was higher than those produced by sucrose (8.78 ± 1.56 g/L) and glucose (9.57 ± 1.96 g/L). Cane molasses favored *B. subtilis* 242 growth. The maximum OD_600_ value of 15.87 ± 0.15 was obtained in the medium with cane molasses, which was higher than those in the medium with sucrose (10.29 ± 1.55) and glucose (6.63 ± 0.49). This suggested that cane molasses could be used by *B. subtilis* 242 to produce γ-PGA. Compared with glucose and sucrose, cane molasses resulted in high cell density, which may account for the high yield of γ-PGA. Our study indicated that cane molasses was more suitable for γ-PGA production. Cane molasses is the by-product of sugar manufacture. It contains large amounts of fermentable sugars, trace elements and inorganic salts, which could be used directly by microorganisms [23]. Therefore, cane molasses has been widely used as cheap carbon source and/or nutritional supplement for fermentation [17]. The sugars in cane molasses are mainly composed of sucrose, glucose, and fructose. Glucose is known as the most efficient carbon source for producing γ-PGA [24]. Sucrose, the main sugar in cane molasses, could also be used by *B. subtilis* 242 to produce γ-PGA (Fig. 3a). γ-PGA polymerization is adenosine triphosphate (ATP) dependent [25]. Microbial γ-PGA is biosynthesized from glutamic acid as substrate. The glutamic acid monomers incorporated in γ-PGA can be derived from the cultivation medium or de novo synthesis from citric acid in the tricarboxylic acid (TCA) cycle [3, 26]. The sugars in cane molasses could be utilized as an energy source and the metabolites of sugars may enter TCA cycle to provide precursors for glutamic acid biosynthesis.

Yeast extract (YE) consists of a mixture of carbohydrates, amino acids, peptides, vitamins, and trace elements. It is important for microbial growth [27]. In this study, the effects of YE on γ-PGA production were detected in the medium with and without YE. The use of YE favored γ-PGA production. γ-PGA concentration and conversion rate of glutamate (*g*_γ-PGA_/*g*_glutamate_) were significantly enhanced with the addition of YE (Fig. 3b). Although cane molasses contains nitrogen source, vitamins, and other growth-promoting factors [28], nitrogen was also needed for microbial growth and metabolism.

### Optimization of nitrogen sources and cane molasses for γ-PGA production

**Fig. 4:**
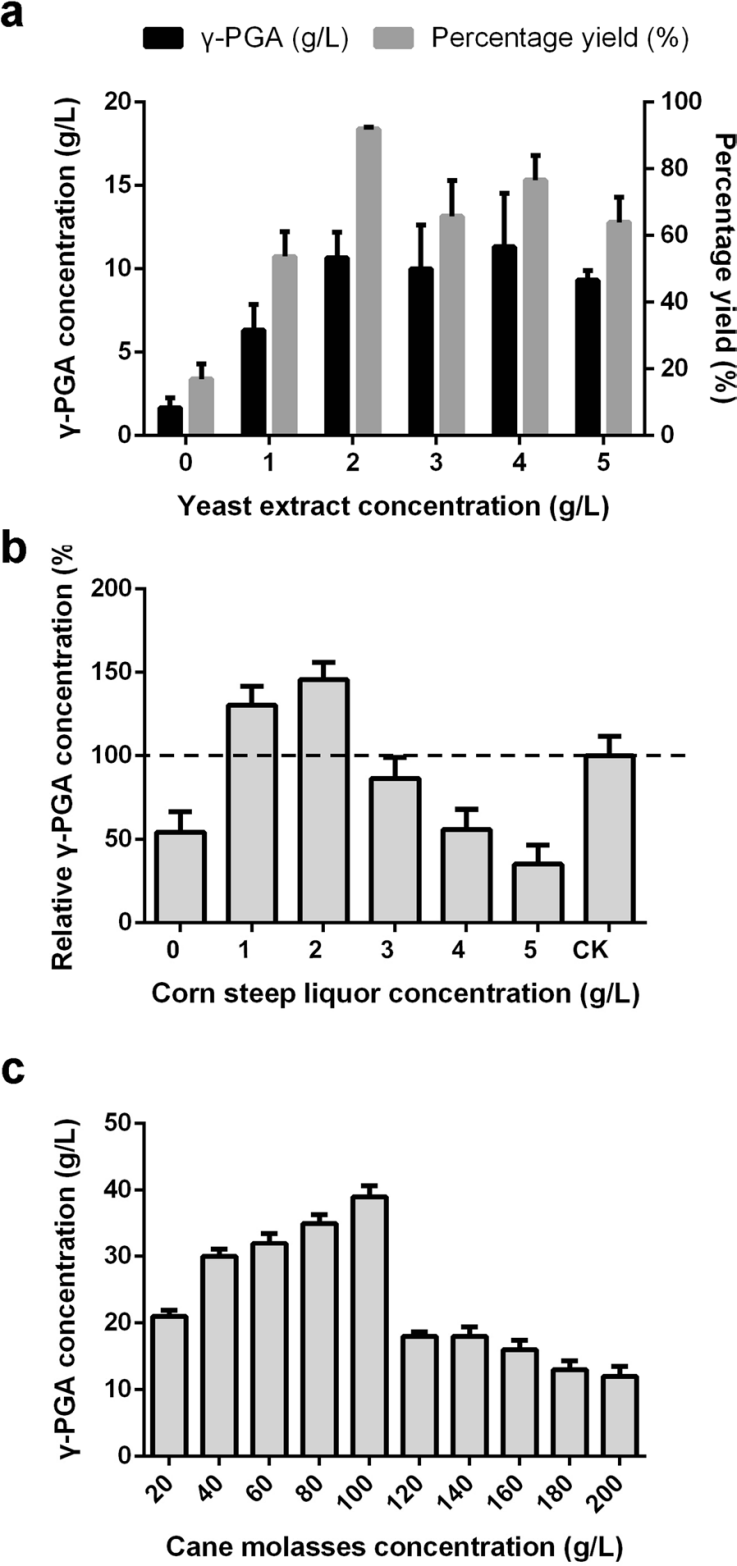
Optimization of fermentation medium. Effects of yeast extract on γ-PGA production (**a**). Effects of corn steep liquor on γ-PGA production (**b**). γ-PGA concentration produced using 2 g/L yeast extract as the sole nitrogen source was defined as 100%. The relative γ-PGA concentration was determined as (*g*_C_/*g*_Y_) × 100%, where *g*_C_ is γ-PGA concentration in the medium with corn steep liquor and *g*_Y_ is γ-PGA concentration in the medium with 2 g/L YE. Effects of cane molasses on γ-PGA production (**c**). Error bars represent SD calculated from three independent determinations

Nitrogen sources are important for γ-PGA production by *B. subtilis* 242. The effect of YE concentration (0, 1, 2, 3, 4 and 5 g/L) on γ-PGA production was studied in flask-shaking fermentation. As shown in Fig. 4a, the γ-PGA concentration increased from 1.67 ± 0.58 to 10.67 ± 1.53 g/L as increasing YE concentration from 0 to 2 g/L. Further increasing the YE concentration (from 3 to 5 g/L) did not significantly increase γ-PGA concentration. The highest percentage yield (*g*_γ-PGA_/*g*_glutamate_, 91.99 ± 0.45%) was obtained at YE concentration of 2 g/L. Although YE favored γ-PGA production, high cost of YE is the main obstacle to the industrial production of γ-PGA. Corn steep liquor is a by-product of the wet milling process and contains crude proteins, amino acids, vitamins, and other nutrients [29]. It has been employed as an inexpensive source of nitrogen for microorganisms in the production of enzymes, antibiotics, and other fermentation products [30]. Different concentrations of corn steep liquor (0, 1, 2, 3, 4 and 5 g/L) were used to investigate the effects of corn steep liquor on γ-PGA production. Considering the highest yield of γ-PGA was obtained with 2 g/L YE (Fig. 4a), the concentration of γ-PGA produced in the medium with 2 g/L YE was set as 100%. The relative concentrations, expressed as percentages of γ-PGA yield in the medium with different concentrations of corn steep liquor relative to that obtained in the medium with 2 g/L YE, was used for the optimization of corn steep liquor. The results in Fig. 4b implied that corn steep liquor was a suitable nitrogen source for γ-PGA synthesis. γ-PGA concentration was enhanced by increasing the concentration corn steep liquor from 1 to 2 g/L. When 2 g/L corn steep liquor was used as the sole nitrogen source, the maximal relative γ-PGA concentration of 145.55 ± 10.41% was achieved, which was 45.55% higher than that obtained from 2 g/L YE. The relative γ-PGA concentration decreased with increasing corn steep liquor concentration from 3 to 5 g/L. Compared with YE, corn steep liquor is an inexpensive nitrogen source that can reduce the amount of YE required for γ-PGA production.

Although cane molasses could be used by *B. subtilis* 242 to produce γ-PGA, the production process of cane molasses will inevitably generate toxic compounds, such as metal ion, 5-hydroxymethylfurfural and others, which could inhibit the growth of *B. subtilis* 242 [17]. To investigate the effects of cane molasses on γ-PGA production, different concentrations of cane molasses were chosen to produce γ-PGA. As shown in Fig. 4c, γ-PGA production was enhanced by increasing cane molasses when cane molasses concentrations were below 100 g/L. The highest γ-PGA concentration of 39 ± 1.70 g/L was obtained in the medium with 100 g/L cane molasses. However, high concentrations of cane molasses (> 100 g/L) decreased γ-PGA production by *B. subtilis* 242. The phenomenon was consistent with the previous reported results that high molasses concentration showed significant inhibition of cell growth and products synthesis [17, 31, 32]. When γ-PGA producers were cultured in medium with high concentration of molasses, the inhibitors in molasses exceeded the tolerance limit of strains [33]. Furthermore, high concentration of sugar may also show inhibitory effects on cell growth [17]. Therefore, cane molasses concentration of 100 g/L was finally selected for γ-PGA fermentation.

### Fed-batch fermentation for γ-PGA production by *B. subtilis* 242

**Fig. 5:**
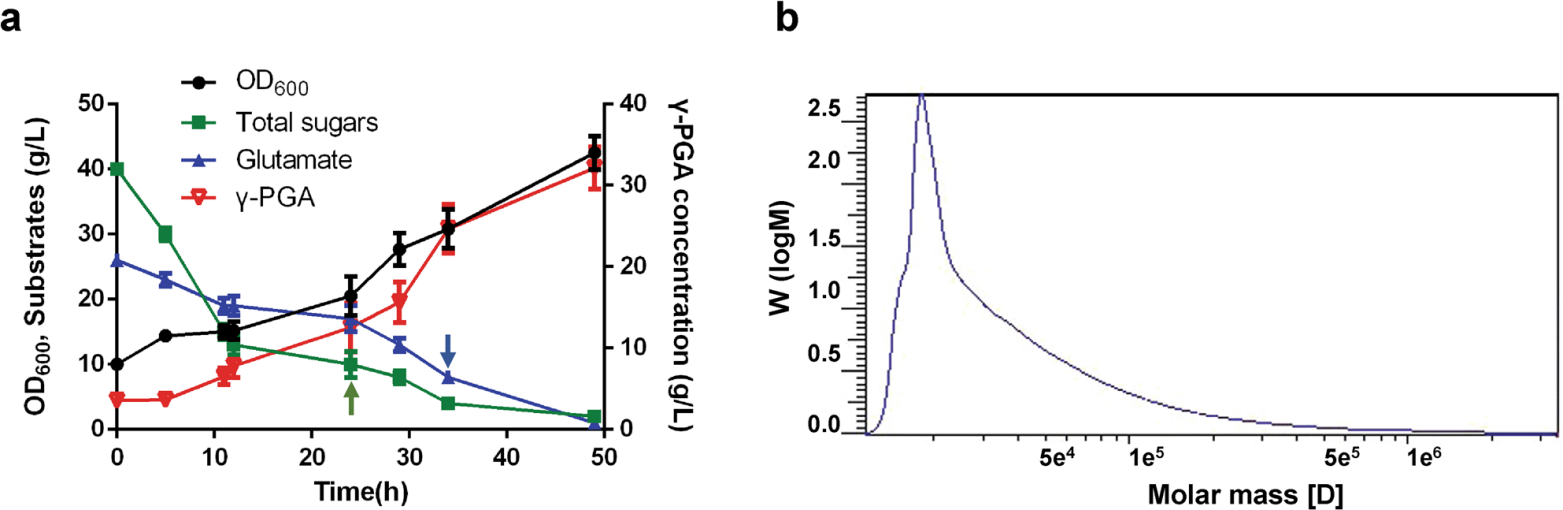
Time profile (**a**) and molecular weight (**b**) of γ-PGA fermentation using cane molasses and corn steep liquor as the carbon and nitrogen sources in a 5-L fermenter. Green arrow: the point at which cane molasses was added. Blue arrow: the point at which glutamate were added

To further investigate the feasibility of industrial γ-PGA production using cane molasses as the substrate, fed-batch fermentation was performed in a 5-L fermentor. A cane molasses concentration of 100 g/L, with the initial sugar concentration of 40 g/L, was used to minimize the inhibition on cell growth and γ-PGA production, then cane molasses was fed into the fermentor at 24 h with a flow rate of 6 mL/h to keep sugar concentration below 10 g/L. As shown in Fig. 5a, the sugars in cane molasses could be utilized by *B. subtilis* 242 to produce γ-PGA. During the first 24 h, small amount of γ-PGA accumulated in the medium. γ-PGA concentration increased after 24 h and finally reached 32.24 g/L with a productivity of 0.67 g/L/h. Glutamate could not only be used as precursor for γ-PGA polymerization, but also could be metabolized as a nitrogen source to maintain cell growth [34]. The initial concentration of glutamate was 26 g/L, and glutamate was fed at 34 h with a flow rate of 2 mL/h to keep glutamate concentration below 5 g/L. The percentage yield (*g*_γ-PGA_/*g*_glutamate_) was 106.39%. The Mw of γ-PGA produced in fed-batch fermentation was 27.99 kDa (Fig. 5b). It has been reported that supplementation with citric acid may improve the synthesis of endogenous glutamic acid, leading to a high yield of γ-PGA [35]. Although exogenous glutamate was added to the medium to biosynthesize γ-PGA, the endogenous glutamic acid derived from TCA cycle could also be used as the precursor of γ-PGA [13]. Therefore, a percentage yield higher than 100% was obtained in fed-batch fermentation.

Several *Bacillus* strains have been reported to produce γ-PGA. However, the Mw of γ-PGA produced by commercially used strains are approximately 1000 kDa, which could not be used for medical applications [36]. Nowadays, γ-PGA with low Mw is mainly produced by degradation of biopolymers using enzymatic, physical, and chemical methods [37]. These methods could not be applied for large-scale production due to high cost during degradation process. In this study, an economical procedure for low-Mw-γ-PGA was obtained by fermentation using cane molasses as the carbon source and corn steep liquor as the nitrogen source. High substrate cost was one of the most important factors limiting γ-PGA production by microbial fermentation. Hitherto, some studies have been done on γ-PGA production using low-cost raw materials as carbon source. Corncob fibers hydrolysates, sugarcane juice and rice straw have been used to produce γ-PGA [14, 38]. Compared with these substrates, cane molasses is an abundant and cheap resource with high sugar concentration (approximately 50%, w/w). The price of cane molasses is $120/t [32], and the price of corn steep liquor is $500–700/t (China Corn Steep Liquor, Corn Steep Liquor Manufacturers, Suppliers, Price | Made-in-China.com). It has been reported that the price of glucose and YE are $520/t and $2500/t, respectively [17]. Therefore, the feedstock cost of γ-PGA production from glucose and YE was estimated to be 1.48 $/kg. If cane molasses and corn steep liquor were used as the carbon and nitrogen sources, the cost would decrease to 0.43 $/kg, which means approximately 70% savings compared to the conventional glucose fermentation (Table 1). Therefore, our study provides an economical approach for γ-PGA production.

**Table 1:** Feedstock cost analysis of γ-PGA production using cane molasses and glucose by *B. subtilis* 242

Culture medium	Carbon source consumed (kg)	Cost ($)	Nitrogen source consumed (kg)	Cost ($)	γ-PGA produced (kg)	Cost ($/kg)	Cost saving^a^ (%)
Glucose + YE	210	109	4	10	80	1.48	–
Cane molasses + corn steep liquor	200	24	4	2	62	0.43	71

^a^The cost analysis includes only carbon and nitrogen sources, not including other components (l-glutamate, K_2_HPO_4_, MgSO_4_.7H_2_O)

## Conclusions

A novel isolated γ-PGA producer, *B. subtilis* 242, was used to produce low-Mw-γ-PGA from cane molasses. After optimization of nitrogen and carbon sources, γ-PGA production of 32.14 g/L γ-PGA with a percentage yield (*g*_γ-PGA_/*g*_glutamate_) of 106.39% was achieved in fed-batch fermentation. The Mw of γ-PGA was 27.99 kDa. This study demonstrated that cane molasses could be used as carbon source for economic γ-PGA production, and provided a sustainable way for low-Mw-γ-PGA from cheap industrial by-product.

## Methods

### Isolation and identification of the new isolated *B. subtilis* 242

*Bacillus subtilis *242 was isolated from the soil in Heilongjiang Province, China. Samples of 1 g were diluted in 100 mL of sterilized distilled water and boiled for 5 min before they were spread on the isolation medium. Luria–Bertani medium (LB; 10 g/L tryptone, 5 g/L YE and 10 g/L NaCl) with 20 g/L l-glutamate was used as the isolation medium. The suspensions were diluted 10^–3^ to 10^–6^, and an aliquot (100 μL) of each suspension was spread on isolation medium. After incubation at 37 °C for 24 h, sticky clones were transferred to 20 mL of basal medium in a 100-mL flask and cultured at 37 °C for 12 h with agitation at 200 rpm. The samples (1%, *v*/*v*) were transferred into 50 mL of medium containing 20 g/L glucose, 20 g/L l-glutamate, 5 g/L YE, 2 g/L K_2_HPO_4_·3H_2_O and 0.25 g/L MgSO_4_·7H_2_O (pH 7.0) in a 300-mL flask, and incubated at 37 °C for 48 h with agitation at 200 rpm. The strain with the highest γ-PGA yield was designated as strain 242, and was stored in LB medium with sterile glycerol (25%, v/v) at − 80 °C for further study.

The 16s rDNA sequence of strain 242 was determined as described in previous study [13]. The partial 16s rDNA region was amplified using the 27F and 1492R universal primers (27F: 5ʹ-AGAGTTTGATCCTGGCTCAG-3ʹ and 1492R: 5ʹ-TACGGCTACCTTGTTACGACTT-3ʹ). The amplified DNA fragments were sequenced and their homology was analyzed using BLAST at the National Center for Biotechnology Information (NCBI) website (BLAST: Basic Local Alignment Search Tool (nih.gov)). The phylogenetic tree was constructed using the neighbor-joining method in MEGA 7.0.

### Total RNA extraction and RT-PCR

Bacterial RNA was extracted from *B. subtilis* 242 cultures at fermentation time of 6 h, 12 h, 24 h, 24 h, and 36 h, respectively. Total RNA was extracted using an E.Z.N.A. bacterial RNA kit (Omega). The total RNA concentration was determined via absorbance at 260 nm (NanoVue spectrophotometer; GE). By using random hexamer primers, cDNA copies were synthesized with a Fast Quant RT kit (with gDNase) (Tiangen, China) and amplified with SYBR Premix Ex Taq (TaKaRa, China) using the LightCylcer 96 RT-PCR detection system (Roche, U.S.A). The specific primers for partial 16s rDNA are q-16s-F: 5ʹ-CACTGGGACTGAGACACGG-3ʹ and q-16s-R: 5ʹ-ACAACGCTTGCCACCTA-3ʹ. The specific primers for *pgdS* are *pgdS*-F: 5ʹ-ACTGGCAAACTGGAAGAA-3ʹ and *pgdS*-R: 5ʹ-CCTGATGGATCGAAACC-3ʹ. Threshold cycles (C_T_) for each PCR with different cDNA concentrations were determined and compared with that for standard DNA (the 16S rRNA gene) analyzed at the same time. The 2^−△△Ct^ relative quantification method was used to determine mRNA levels [39]. Results reported are the averages of four experiments with a variability of < 15%.

### Media and culture conditions

LB medium was used as the basal medium for the pre-culture of *B. subtilis* 242. For γ-PGA fermentation, *B. subtilis* 242 was inoculated into a 500 mL flask with 50 mL of seed medium containing 20 g/L glucose, 20 g/L l-glutamate, 5 g/L YE, 2 g/L K_2_HPO_4_·3H_2_O and 0.25 g/L MgSO_4_·7H_2_O (pH 7.0). Cells were grown at 37 °C for 16 h with shaking at 200 rpm. Subsequently, the seed cells were inoculated into the fermentation medium containing 40 g/L l-glutamate, 2 g/L K_2_HPO_4_·3H_2_O, 0.25 g/L MgSO_4_·7H_2_O, carbon sources (glucose, sucrose, or cane molasses), and nitrogen sources (YE or corn steep liquor). The inoculation was 10% (v/v) and pH value was adjusted to 7.0. The cells were cultured at 37 °C, 200 rpm. The cane molasses was a gift from Prof. Nengzhong Xie, Guangxi Academy of Sciences, Guangxi, China. Corn seep liquor was purchased from Weiduofeng Biotechnology Co., Ltd (Shandong, China).

### Optimization of nitrogen sources and cane molasses for γ-PGA production

The seed culture of *B. subtilis* 242 was inoculated into fermentation medium and cultivated at 37 °C and 220 rpm for 48 h. To investigate the feasibility of cane molasses utilization for γ-PGA production, 40 g/L cane molasses (containing ~ 20 g/L total sugars), 20 g/L sucrose, and 20 g/L glucose were added, individually, to the fermentation medium. To investigate the effects of YE on γ-PGA production, 40 g/L cane molasses with or without 5 g/L YE were added to the fermentation medium. To optimize YE concentration, 0, 1 g/L, 2 g/L, 3 g/L, 4 g/L, and 5 g/L YE were added to the fermentation medium. To investigate the effects of corn steep liquor on γ-PGA production, 0, 1 g/L, 2 g/L, 3 g/L, 4 g/L, and 5 g/L corn steep liquor were added to the fermentation medium. The concentration of γ-PGA produced in the medium with 2 g/L YE was defined as 100%. The relative γ-PGA concentration was determined as (*g*_C_/*g*_Y_) × 100%, where *g*_C_ is γ-PGA concentration in the medium with corn steep liquor and *g*_Y_ is γ-PGA concentration in the medium with 2 g/L YE. To optimize cane molasses concentration, 20 g/L, 40 g/L, 60 g/L, 80 g/L, 100 g/L, 120 g/L, 140 g/L, 160 g/L, 180 g/L, and 200 g/L cane molasses were added to the fermentation medium.

### Fed-batch γ-PGA fermentation

Fed-batch fermentation of *B. subtilis* 242 was carried out in a 5-L fermenter (Baoxing, Shanghai, China) containing 2 L of medium. The optimized medium comprised 100 g/L cane molasses (containing 40 g/L sugars), 30 g/L l-glutamate, 2 g/L corn steep liquor, 2 g/L K_2_HPO_4_, and 0.25 g/L MgSO_4_. Cultivation was carried out at 37 °C with an airflow of 1.5 vvm. Dissolved oxygen level was maintained at 10% by adjusting the agitation rate from 200 to 600 rpm. NH_4_OH was added automatically to maintain the pH at 7.0. Cane molasses (300 g/L) and l-glutamate (400 g/L) were added to the fermentor with a flow rate of 6 mL/h and 2 mL/h, respectively, to keep the concentrations of sugars and l-glutamate lower than 10 g/L and 5 g/L, respectively. Samples were collected periodically to determine OD_600_, total sugars, l-glutamate, and γ-PGA.

### Analytical methods

Sugars (sucrose, fructose and glucose) in cane molasses were determined by HPLC (Agilent Technologies 1260 Infinity Series, USA) with an Aminex HPX-87H Column (300 × 7.8 mm) (Bio-Rad) and differential refractive index detector (RID). The mobile phase was 6 mM H_2_SO_4_ and flow rate was 0.5 mL/min. The purification of γ-PGA was carried out by the method reported previously [40]. γ-PGA was hydrolyzed using 6 M HCl under vacuum at 120 °C for 12 h. The glutamate generated were analyzed by HPLC with Eclipse Plus C18 (4.6 × 250 mm, Agilent) [41]. γ-PGA Mw was determined by GPC [42]. Samples were analyzed on an Agilent 1100 HPLC System, equipped with Ultrahydrogel TM 2000 column, Ultrahydrogel TM 250 column, Ultrahydrogel TM 120 column (7.8 × 300 mm, Waters, USA) and a refractive index (RI) detector. Pullulan standards of narrow polydispersity (SHANGHAI ZZBIO CO., Ltd., Shanghai, China) were employed to establish a calibration curve. During fed-batch fermentation, l-glutamate were monitored by SBA-40C bioanalyzer (Academy of Sciences, Shandong, China). Cell growth was monitored by measuring the OD_600_ of the culture broth using the 752N spectrophotometer (Shanghai Opler Instrument Co., Ltd., Shanghai, China).

### Statistical analysis

All tests were repeated at least three times and the data were expressed as mean ± SD. Data were analyzed by SPSS Statistics software v.19.0. Pearson correlation coefficient, t-test, and ANOVA test were carried out to compare means values and p < 0.05 were considered statistically significant.

## Data Availability

All data generated or analyzed during this study are included in this article.
